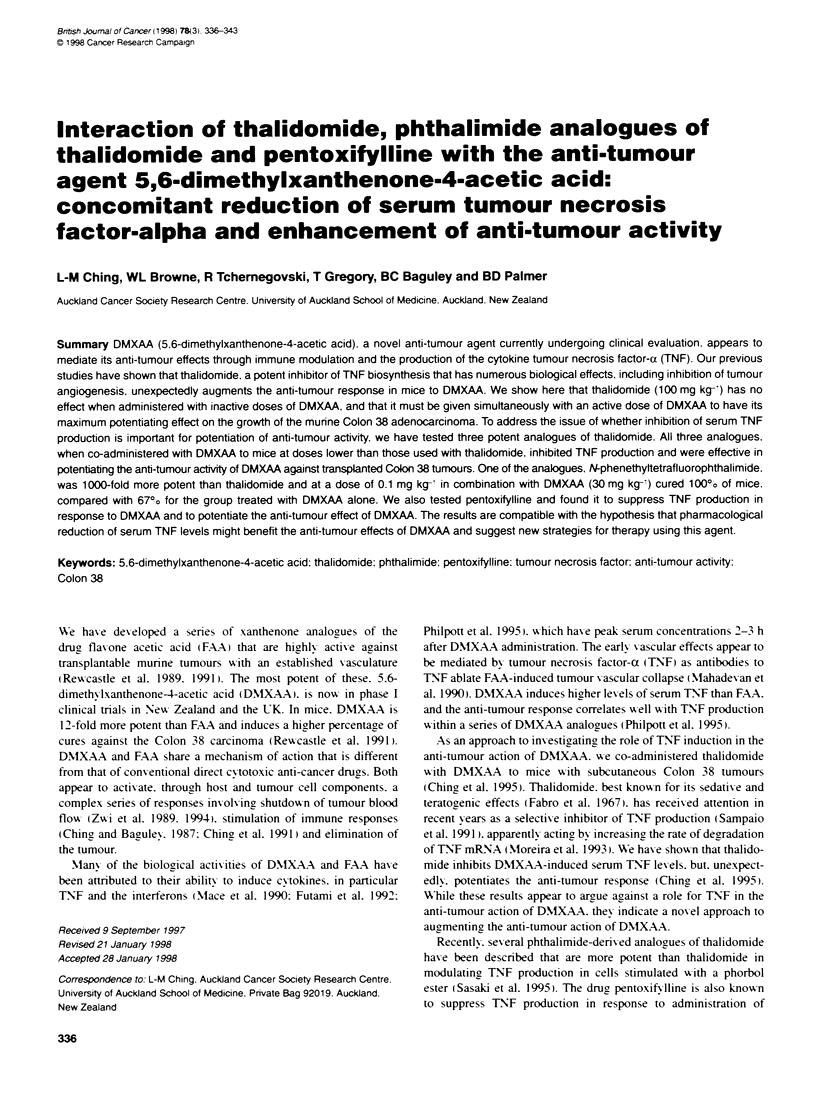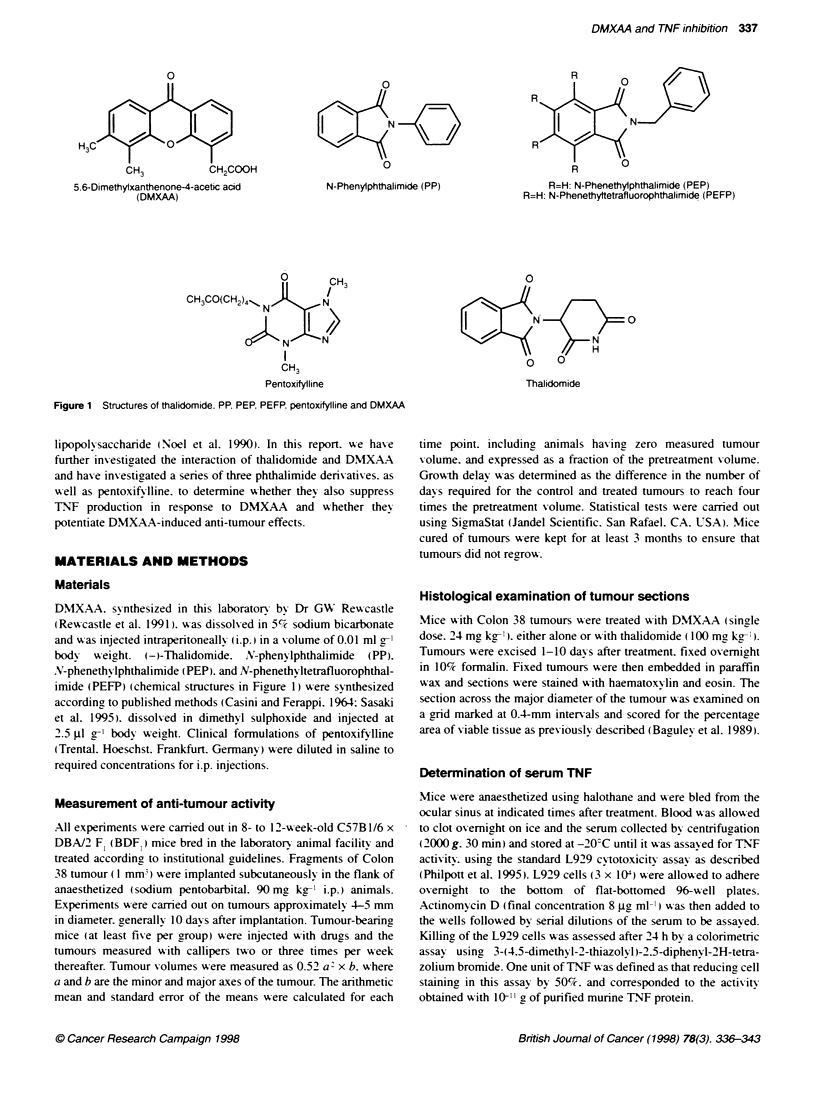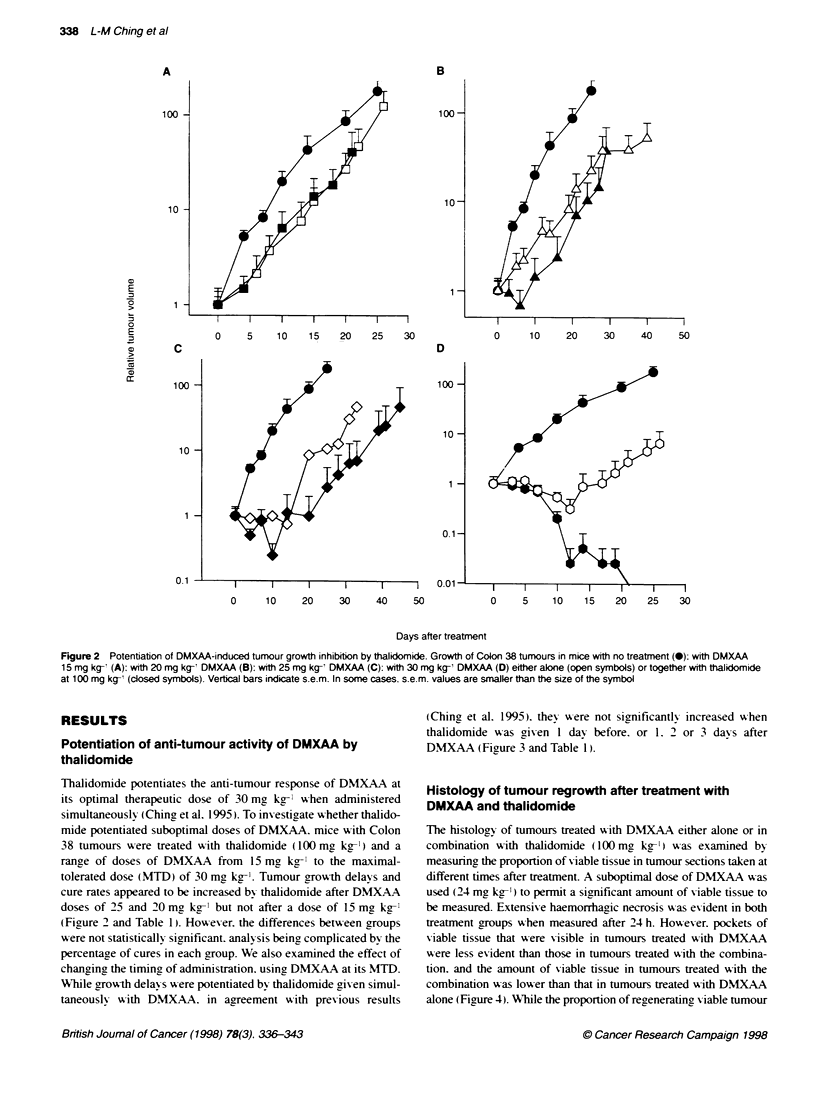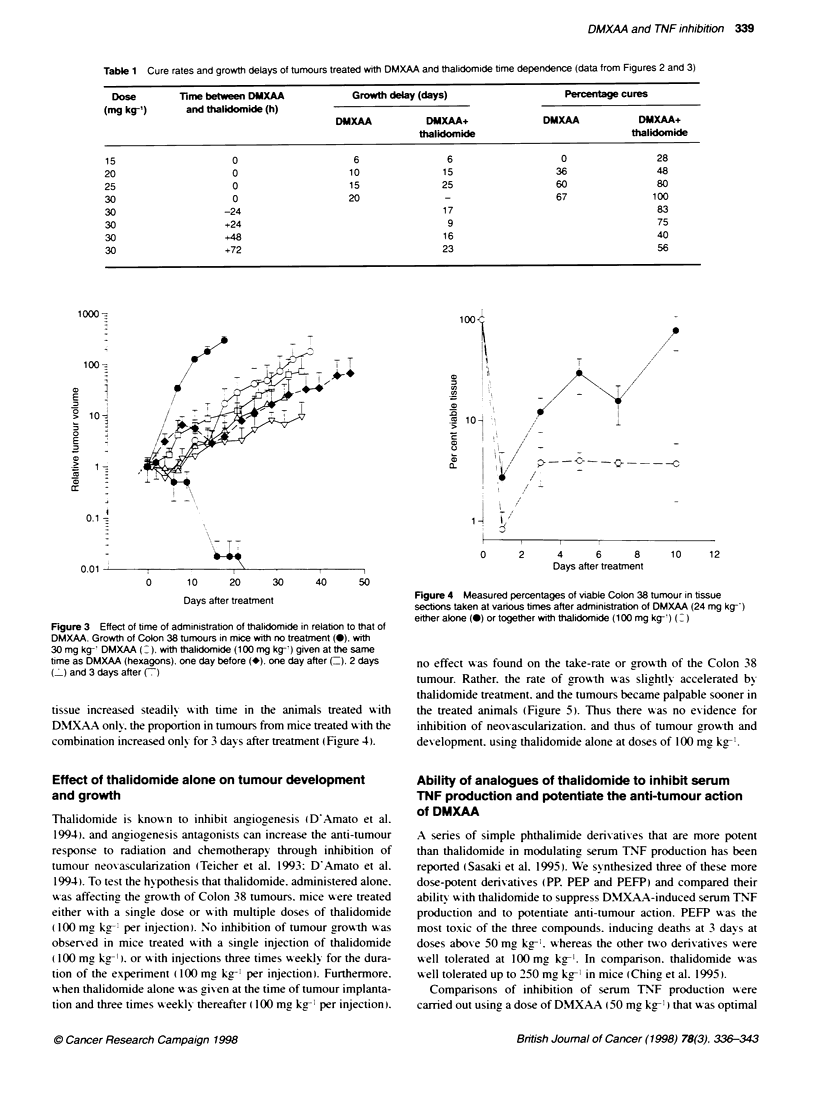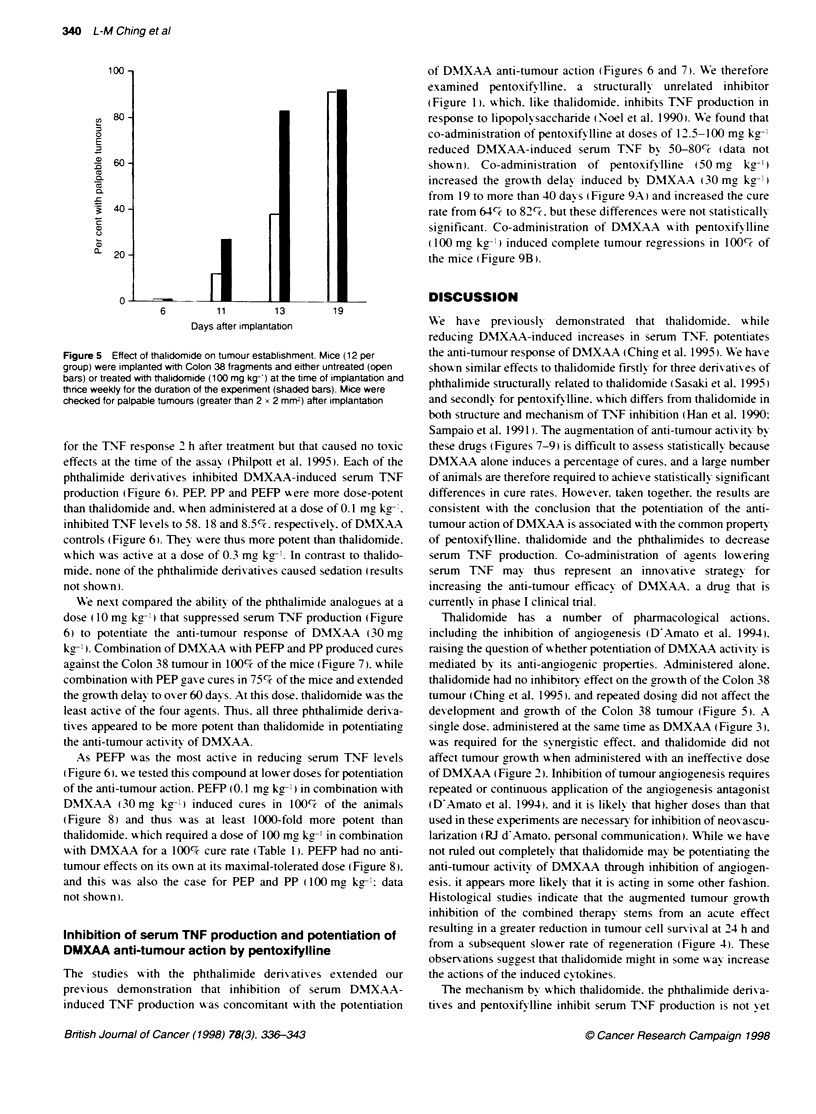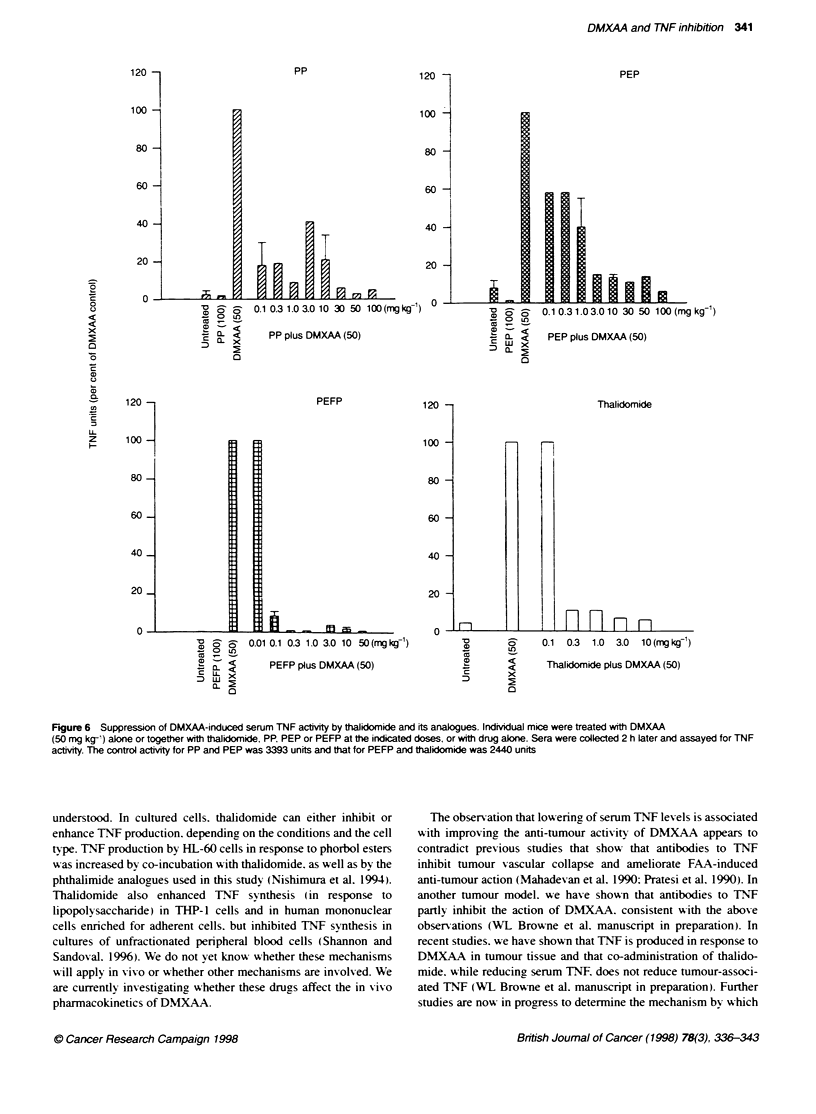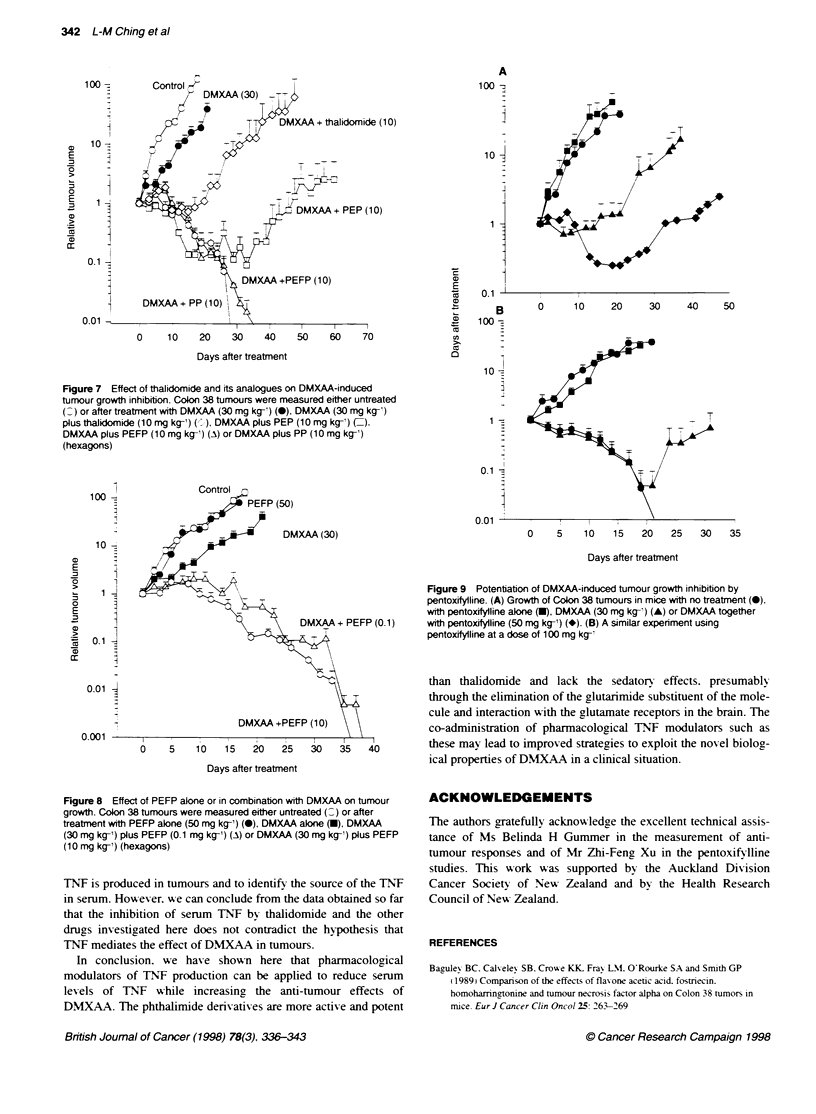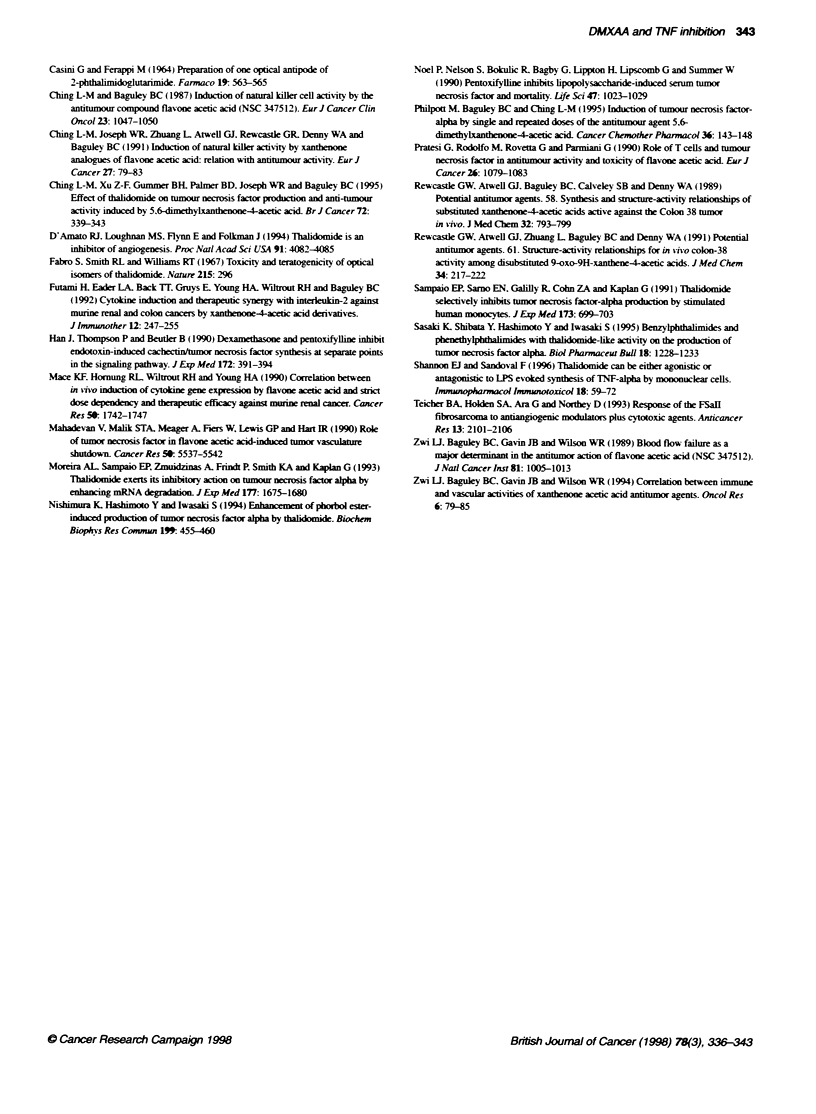# Interaction of thalidomide, phthalimide analogues of thalidomide and pentoxifylline with the anti-tumour agent 5,6-dimethylxanthenone-4-acetic acid: concomitant reduction of serum tumour necrosis factor-alpha and enhancement of anti-tumour activity.

**DOI:** 10.1038/bjc.1998.495

**Published:** 1998-08

**Authors:** L. M. Ching, W. L. Browne, R. Tchernegovski, T. Gregory, B. C. Baguley, B. D. Palmer

**Affiliations:** Auckland Cancer Society Research Centre, University of Auckland School of Medicine, New Zealand.

## Abstract

DMXAA (5,6-dimethylxanthenone-4-acetic acid), a novel anti-tumour agent currently undergoing clinical evaluation, appears to mediate its anti-tumour effects through immune modulation and the production of the cytokine tumour necrosis factor-alpha (TNF). Our previous studies have shown that thalidomide, a potent inhibitor of TNF biosynthesis that has numerous biological effects, including inhibition of tumour angiogenesis, unexpectedly augments the anti-tumour response in mice to DMXAA. We show here that thalidomide (100 mg kg(-1)) has no effect when administered with inactive doses of DMXAA, and that it must be given simultaneously with an active dose of DMXAA to have its maximum potentiating effect on the growth of the murine Colon 38 adenocarcinoma. To address the issue of whether inhibition of serum TNF production is important for potentiation of anti-tumour activity, we have tested three potent analogues of thalidomide. All three analogues, when co-administered with DMXAA to mice at doses lower than those used with thalidomide, inhibited TNF production and were effective in potentiating the anti-tumour activity of DMXAA against transplanted Colon 38 tumours. One of the analogues, N-phenethyltetrafluorophthalimide, was 1000-fold more potent than thalidomide and at a dose of 0.1 mg kg(-1) in combination with DMXAA (30 mg kg(-1)) cured 100% of mice, compared with 67% for the group treated with DMXAA alone. We also tested pentoxifylline and found it to suppress TNF production in response to DMXAA and to potentiate the anti-tumour effect of DMXAA. The results are compatible with the hypothesis that pharmacological reduction of serum TNF levels might benefit the anti-tumour effects of DMXAA and suggest new strategies for therapy using this agent.


British Journal of Cancer (1998) 78(3). 336–343

© 1998 Cancer Research Campaign

### Interaction of thalidomide, phthalimide analogues of thalidomide and pentoxifylline with the anti-tumour agent 5,6-dimethylxanthenone-4-acetic acid: concomitant reduction of serum tumour necrosis factor-alpha and enhancement of anti-tumour activity

**L-M Ching, WL Browne, R Tchernegovski, T Gregory, BC Baguley and BD Palmer**

Auckland Cancer Society Research Centre. University of Auckland School of Medicine. Auckland. New Zealand

**Summary** DMXAA (5,6-dimethylxanthenone-4-acetic acid), a novel anti-tumour agent currently undergoing clinical evaluation, appears to mediate its anti-tumour effects through immune modulation and the production of the cytokine tumour necrosis factor-α (TNF). Our previous studies have shown that thalidomide, a potent inhibitor of TNF biosynthesis that has numerous biological effects, including inhibition of tumour angiogenesis, unexpectedly augments the anti-tumour response in mice to DMXAA. We show here that thalidomide (100 mg kg$^{-1}$) has no effect when administered with inactive doses of DMXAA, and that it must be given simultaneously with an active dose of DMXAA to have its maximum potentiating effect on the growth of the murine Colon 38 adenocarcinoma. To address the issue of whether inhibition of serum TNF production is important for potentiation of anti-tumour activity, we have tested three potent analogues of thalidomide. All three analogues, when co-administered with DMXAA to mice at doses lower than those used with thalidomide, inhibited TNF production and were effective in potentiating the anti-tumour activity of DMXAA against transplanted Colon 38 tumours. One of the analogues, *N*-phenethyltetrafluorophthalimide, was 1000-fold more potent than thalidomide and at a dose of 0.1 mg kg$^{-1}$ in combination with DMXAA (30 mg kg$^{-1}$) cured 100% of mice, compared with 67% for the group treated with DMXAA alone. We also tested pentoxifylline and found it to suppress TNF production in response to DMXAA and to potentiate the anti-tumour effect of DMXAA. The results are compatible with the hypothesis that pharmacological reduction of serum TNF levels might benefit the anti-tumour effects of DMXAA and suggest new strategies for therapy using this agent.

**Keywords:** 5,6-dimethylxanthenone-4-acetic acid; thalidomide; phthalimide; pentoxifylline; tumour necrosis factor; anti-tumour activity; Colon 38

We have developed a series of xanthenone analogues of the drug flavone acetic acid (FAA) that are highly active against transplantable murine tumours with an established vasculature (Rewcastle et al. 1989, 1991). The most potent of these, 5,6-dimethylxanthenone-4-acetic acid (DMXAA), is now in phase I clinical trials in New Zealand and the UK. In mice, DMXAA is 12-fold more potent than FAA and induces a higher percentage of cures against the Colon 38 carcinoma (Rewcastle et al. 1991). DMXAA and FAA share a mechanism of action that is different from that of conventional direct cytotoxic anti-cancer drugs. Both appear to activate, through host and tumour cell components, a complex series of responses involving shutdown of tumour blood flow (Zwi et al. 1989, 1994), stimulation of immune responses (Ching and Baguley, 1987; Ching et al. 1991) and elimination of the tumour.

Many of the biological activities of DMXAA and FAA have been attributed to their ability to induce cytokines, in particular TNF and the interferons (Mace et al. 1990; Futami et al. 1992; Philpott et al. 1995), which have peak serum concentrations 2–3 h after DMXAA administration. The early vascular effects appear to be mediated by tumour necrosis factor-α (TNF) as antibodies to TNF ablate FAA-induced tumour vascular collapse (Mahadevan et al. 1990). DMXAA induces higher levels of serum TNF than FAA, and the anti-tumour response correlates well with TNF production within a series of DMXAA analogues (Philpott et al. 1995).

As an approach to investigating the role of TNF induction in the anti-tumour action of DMXAA, we co-administered thalidomide with DMXAA to mice with subcutaneous Colon 38 tumours (Ching et al. 1995). Thalidomide, best known for its sedative and teratogenic effects (Fabro et al. 1967), has received attention in recent years as a selective inhibitor of TNF production (Sampaio et al. 1991), apparently acting by increasing the rate of degradation of TNF mRNA (Moreira et al. 1993). We have shown that thalidomide inhibits DMXAA-induced serum TNF levels, but, unexpectedly, potentiates the anti-tumour response (Ching et al. 1995). While these results appear to argue against a role for TNF in the anti-tumour action of DMXAA, they indicate a novel approach to augmenting the anti-tumour action of DMXAA.

Recently, several phthalimide-derived analogues of thalidomide have been described that are more potent than thalidomide in modulating TNF production in cells stimulated with a phorbol ester (Sasaki et al. 1995). The drug pentoxifylline is also known to suppress TNF production in response to administration of

*Received 9 September 1997*
*Revised 21 January 1998*
*Accepted 28 January 1998*

*Correspondence to:* L-M Ching. Auckland Cancer Society Research Centre. University of Auckland School of Medicine. Private Bag 92019. Auckland. New Zealand

**Figure 1** Structures of thalidomide, PP, PEP, PEFP, pentoxifylline and DMXAA

lipopolysaccharide (Noel et al. 1990). In this report, we have further investigated the interaction of thalidomide and DMXAA and have investigated a series of three phthalimide derivatives, as well as pentoxifylline, to determine whether they also suppress TNF production in response to DMXAA and whether they potentiate DMXAA-induced anti-tumour effects.

### MATERIALS AND METHODS

### Materials

DMXAA, synthesized in this laboratory by Dr GW Rewcastle (Rewcastle et al. 1991), was dissolved in 5% sodium bicarbonate and was injected intraperitoneally (i.p.) in a volume of 0.01 ml $g^{-1}$ body weight. (–)-Thalidomide, *N*-phenylphthalimide (PP), *N*-phenethylphthalimide (PEP), and *N*-phenethyltetrafluorophthalimide (PEFP) (chemical structures in Figure 1) were synthesized according to published methods (Casini and Ferappi, 1964; Sasaki et al. 1995), dissolved in dimethyl sulphoxide and injected at 2.5 μl $g^{-1}$ body weight. Clinical formulations of pentoxifylline (Trental, Hoeschst, Frankfurt, Germany) were diluted in saline to required concentrations for i.p. injections.

### Measurement of anti-tumour activity

All experiments were carried out in 8- to 12-week-old C57B1/6 × DBA/2 $F_1$ ($BDF_1$) mice bred in the laboratory animal facility and treated according to institutional guidelines. Fragments of Colon 38 tumour (1 $mm^3$) were implanted subcutaneously in the flank of anaesthetized (sodium pentobarbital, 90 mg $kg^{-1}$ i.p.) animals. Experiments were carried out on tumours approximately 4–5 mm in diameter, generally 10 days after implantation. Tumour-bearing mice (at least five per group) were injected with drugs and the tumours measured with callipers two or three times per week thereafter. Tumour volumes were measured as $0.52\ a^2 \times b$, where $a$ and $b$ are the minor and major axes of the tumour. The arithmetic mean and standard error of the means were calculated for each time point, including animals having zero measured tumour volume, and expressed as a fraction of the pretreatment volume. Growth delay was determined as the difference in the number of days required for the control and treated tumours to reach four times the pretreatment volume. Statistical tests were carried out using SigmaStat (Jandel Scientific, San Rafael, CA, USA). Mice cured of tumours were kept for at least 3 months to ensure that tumours did not regrow.

### Histological examination of tumour sections

Mice with Colon 38 tumours were treated with DMXAA (single dose, 24 mg $kg^{-1}$), either alone or with thalidomide (100 mg $kg^{-1}$). Tumours were excised 1–10 days after treatment, fixed overnight in 10% formalin. Fixed tumours were then embedded in paraffin wax and sections were stained with haematoxylin and eosin. The section across the major diameter of the tumour was examined on a grid marked at 0.4-mm intervals and scored for the percentage area of viable tissue as previously described (Baguley et al. 1989).

### Determination of serum TNF

Mice were anaesthetized using halothane and were bled from the ocular sinus at indicated times after treatment. Blood was allowed to clot overnight on ice and the serum collected by centrifugation (2000 *g*, 30 min) and stored at –20°C until it was assayed for TNF activity, using the standard L929 cytotoxicity assay as described (Philpott et al. 1995). L929 cells ($3 \times 10^4$) were allowed to adhere overnight to the bottom of flat-bottomed 96-well plates. Actinomycin D (final concentration 8 μg $ml^{-1}$) was then added to the wells followed by serial dilutions of the serum to be assayed. Killing of the L929 cells was assessed after 24 h by a colorimetric assay using 3-(4,5-dimethyl-2-thiazolyl)-2,5-diphenyl-2H-tetrazolium bromide. One unit of TNF was defined as that reducing cell staining in this assay by 50%, and corresponded to the activity obtained with $10^{-11}$ g of purified murine TNF protein.

© Cancer Research Campaign 1998

**Figure 2** Potentiation of DMXAA-induced tumour growth inhibition by thalidomide. Growth of Colon 38 tumours in mice with no treatment (●); with DMXAA 15 mg kg⁻¹ (**A**); with 20 mg kg⁻¹ DMXAA (**B**); with 25 mg kg⁻¹ DMXAA (**C**); with 30 mg kg⁻¹ DMXAA (**D**) either alone (open symbols) or together with thalidomide at 100 mg kg⁻¹ (closed symbols). Vertical bars indicate s.e.m. In some cases, s.e.m. values are smaller than the size of the symbol

### RESULTS

### Potentiation of anti-tumour activity of DMXAA by thalidomide

Thalidomide potentiates the anti-tumour response of DMXAA at its optimal therapeutic dose of 30 mg $kg^{-1}$ when administered simultaneously (Ching et al, 1995). To investigate whether thalidomide potentiated suboptimal doses of DMXAA, mice with Colon 38 tumours were treated with thalidomide (100 mg $kg^{-1}$) and a range of doses of DMXAA from 15 mg $kg^{-1}$ to the maximal-tolerated dose (MTD) of 30 mg $kg^{-1}$. Tumour growth delays and cure rates appeared to be increased by thalidomide after DMXAA doses of 25 and 20 mg $kg^{-1}$ but not after a dose of 15 mg $kg^{-1}$ (Figure 2 and Table 1). However, the differences between groups were not statistically significant, analysis being complicated by the percentage of cures in each group. We also examined the effect of changing the timing of administration, using DMXAA at its MTD. While growth delays were potentiated by thalidomide given simultaneously with DMXAA, in agreement with previous results (Ching et al, 1995), they were not significantly increased when thalidomide was given 1 day before, or 1, 2 or 3 days after DMXAA (Figure 3 and Table 1).

### Histology of tumour regrowth after treatment with DMXAA and thalidomide

The histology of tumours treated with DMXAA either alone or in combination with thalidomide (100 mg $kg^{-1}$) was examined by measuring the proportion of viable tissue in tumour sections taken at different times after treatment. A suboptimal dose of DMXAA was used (24 mg $kg^{-1}$) to permit a significant amount of viable tissue to be measured. Extensive haemorrhagic necrosis was evident in both treatment groups when measured after 24 h. However, pockets of viable tissue that were visible in tumours treated with DMXAA were less evident than those in tumours treated with the combination, and the amount of viable tissue in tumours treated with the combination was lower than that in tumours treated with DMXAA alone (Figure 4). While the proportion of regenerating viable tumour

 © Cancer Research Campaign 1998

Table 1 Cure rates and growth delays of tumours treated with DMXAA and thalidomide time dependence (data from Figures 2 and 3)

| Dose (mg kg$^{-1}$) | Time between DMXAA and thalidomide (h) | Growth delay (days) | | Percentage cures | |
|---|---|---|---|---|---|
| | | DMXAA | DMXAA+ thalidomide | DMXAA | DMXAA+ thalidomide |
| 15 | 0 | 6 | 6 | 0 | 28 |
| 20 | 0 | 10 | 15 | 36 | 48 |
| 25 | 0 | 15 | 25 | 60 | 80 |
| 30 | 0 | 20 | – | 67 | 100 |
| 30 | –24 | | 17 | | 83 |
| 30 | +24 | | 9 | | 75 |
| 30 | +48 | | 16 | | 40 |
| 30 | +72 | | 23 | | 56 |

Figure 3 Effect of time of administration of thalidomide in relation to that of DMXAA. Growth of Colon 38 tumours in mice with no treatment (●), with 30 mg kg$^{-1}$ DMXAA (○), with thalidomide (100 mg kg$^{-1}$) given at the same time as DMXAA (hexagons), one day before (◆), one day after (□), 2 days (△) and 3 days after (▽)

Figure 4 Measured percentages of viable Colon 38 tumour in tissue sections taken at various times after administration of DMXAA (24 mg kg$^{-1}$) either alone (●) or together with thalidomide (100 mg kg$^{-1}$) (○)

tissue increased steadily with time in the animals treated with DMXAA only, the proportion in tumours from mice treated with the combination increased only for 3 days after treatment (Figure 4).

### Effect of thalidomide alone on tumour development and growth

Thalidomide is known to inhibit angiogenesis (D'Amato et al, 1994), and angiogenesis antagonists can increase the anti-tumour response to radiation and chemotherapy through inhibition of tumour neovascularization (Teicher et al, 1993; D'Amato et al, 1994). To test the hypothesis that thalidomide, administered alone, was affecting the growth of Colon 38 tumours, mice were treated either with a single dose or with multiple doses of thalidomide (100 mg kg$^{-1}$ per injection). No inhibition of tumour growth was observed in mice treated with a single injection of thalidomide (100 mg kg$^{-1}$), or with injections three times weekly for the duration of the experiment (100 mg kg$^{-1}$ per injection). Furthermore, when thalidomide alone was given at the time of tumour implantation and three times weekly thereafter (100 mg kg$^{-1}$ per injection), no effect was found on the take-rate or growth of the Colon 38 tumour. Rather, the rate of growth was slightly accelerated by thalidomide treatment, and the tumours became palpable sooner in the treated animals (Figure 5). Thus there was no evidence for inhibition of neovascularization, and thus of tumour growth and development, using thalidomide alone at doses of 100 mg kg$^{-1}$.

### Ability of analogues of thalidomide to inhibit serum TNF production and potentiate the anti-tumour action of DMXAA

A series of simple phthalimide derivatives that are more potent than thalidomide in modulating serum TNF production has been reported (Sasaki et al, 1995). We synthesized three of these more dose-potent derivatives (PP, PEP and PEFP) and compared their ability with thalidomide to suppress DMXAA-induced serum TNF production and to potentiate anti-tumour action. PEFP was the most toxic of the three compounds, inducing deaths at 3 days at doses above 50 mg kg$^{-1}$, whereas the other two derivatives were well tolerated at 100 mg kg$^{-1}$. In comparison, thalidomide was well tolerated up to 250 mg kg$^{-1}$ in mice (Ching et al, 1995).

Comparisons of inhibition of serum TNF production were carried out using a dose of DMXAA (50 mg kg$^{-1}$) that was optimal

**Figure 5** Effect of thalidomide on tumour establishment. Mice (12 per group) were implanted with Colon 38 fragments and either untreated (open bars) or treated with thalidomide (100 mg kg⁻¹) at the time of implantation and thrice weekly for the duration of the experiment (shaded bars). Mice were checked for palpable tumours (greater than 2 × 2 mm²) after implantation for the TNF response 2 h after treatment but that caused no toxic effects at the time of the assay (Philpott et al. 1995). Each of the phthalimide derivatives inhibited DMXAA-induced serum TNF production (Figure 6). PEP, PP and PEFP were more dose-potent than thalidomide and, when administered at a dose of 0.1 mg $kg^{-1}$, inhibited TNF levels to 58, 18 and 8.5%, respectively, of DMXAA controls (Figure 6). They were thus more potent than thalidomide, which was active at a dose of 0.3 mg $kg^{-1}$. In contrast to thalidomide, none of the phthalimide derivatives caused sedation (results not shown).

We next compared the ability of the phthalimide analogues at a dose (10 mg $kg^{-1}$) that suppressed serum TNF production (Figure 6) to potentiate the anti-tumour response of DMXAA (30 mg $kg^{-1}$). Combination of DMXAA with PEFP and PP produced cures against the Colon 38 tumour in 100% of the mice (Figure 7), while combination with PEP gave cures in 75% of the mice and extended the growth delay to over 60 days. At this dose, thalidomide was the least active of the four agents. Thus, all three phthalimide derivatives appeared to be more potent than thalidomide in potentiating the anti-tumour activity of DMXAA.

As PEFP was the most active in reducing serum TNF levels (Figure 6), we tested this compound at lower doses for potentiation of the anti-tumour action. PEFP (0.1 mg $kg^{-1}$) in combination with DMXAA (30 mg $kg^{-1}$) induced cures in 100% of the animals (Figure 8) and thus was at least 1000-fold more potent than thalidomide, which required a dose of 100 mg $kg^{-1}$ in combination with DMXAA for a 100% cure rate (Table 1). PEFP had no anti-tumour effects on its own at its maximal-tolerated dose (Figure 8), and this was also the case for PEP and PP (100 mg $kg^{-1}$; data not shown).

### Inhibition of serum TNF production and potentiation of DMXAA anti-tumour action by pentoxifylline

The studies with the phthalimide derivatives extended our previous demonstration that inhibition of serum DMXAA-induced TNF production was concomitant with the potentiation of DMXAA anti-tumour action (Figures 6 and 7). We therefore examined pentoxifylline, a structurally unrelated inhibitor (Figure 1), which, like thalidomide, inhibits TNF production in response to lipopolysaccharide (Noel et al. 1990). We found that co-administration of pentoxifylline at doses of 12.5–100 mg $kg^{-1}$ reduced DMXAA-induced serum TNF by 50–80% (data not shown). Co-administration of pentoxifylline (50 mg $kg^{-1}$) increased the growth delay induced by DMXAA (30 mg $kg^{-1}$) from 19 to more than 40 days (Figure 9A) and increased the cure rate from 64% to 82%, but these differences were not statistically significant. Co-administration of DMXAA with pentoxifylline (100 mg $kg^{-1}$) induced complete tumour regressions in 100% of the mice (Figure 9B).

### DISCUSSION

We have previously demonstrated that thalidomide, while reducing DMXAA-induced increases in serum TNF, potentiates the anti-tumour response of DMXAA (Ching et al. 1995). We have shown similar effects to thalidomide firstly for three derivatives of phthalimide structurally related to thalidomide (Sasaki et al. 1995) and secondly for pentoxifylline, which differs from thalidomide in both structure and mechanism of TNF inhibition (Han et al. 1990; Sampaio et al. 1991). The augmentation of anti-tumour activity by these drugs (Figures 7–9) is difficult to assess statistically because DMXAA alone induces a percentage of cures, and a large number of animals are therefore required to achieve statistically significant differences in cure rates. However, taken together, the results are consistent with the conclusion that the potentiation of the anti-tumour action of DMXAA is associated with the common property of pentoxifylline, thalidomide and the phthalimides to decrease serum TNF production. Co-administration of agents lowering serum TNF may thus represent an innovative strategy for increasing the anti-tumour efficacy of DMXAA, a drug that is currently in phase I clinical trial.

Thalidomide has a number of pharmacological actions, including the inhibition of angiogenesis (D'Amato et al. 1994), raising the question of whether potentiation of DMXAA activity is mediated by its anti-angiogenic properties. Administered alone, thalidomide had no inhibitory effect on the growth of the Colon 38 tumour (Ching et al. 1995), and repeated dosing did not affect the development and growth of the Colon 38 tumour (Figure 5). A single dose, administered at the same time as DMXAA (Figure 3), was required for the synergistic effect, and thalidomide did not affect tumour growth when administered with an ineffective dose of DMXAA (Figure 2). Inhibition of tumour angiogenesis requires repeated or continuous application of the angiogenesis antagonist (D'Amato et al. 1994), and it is likely that higher doses than that used in these experiments are necessary for inhibition of neovascularization (RJ d'Amato, personal communication). While we have not ruled out completely that thalidomide may be potentiating the anti-tumour activity of DMXAA through inhibition of angiogenesis, it appears more likely that it is acting in some other fashion. Histological studies indicate that the augmented tumour growth inhibition of the combined therapy stems from an acute effect resulting in a greater reduction in tumour cell survival at 24 h and from a subsequent slower rate of regeneration (Figure 4). These observations suggest that thalidomide might in some way increase the actions of the induced cytokines.

The mechanism by which thalidomide, the phthalimide derivatives and pentoxifylline inhibit serum TNF production is not yet

 © Cancer Research Campaign 1998

**Figure 6** Suppression of DMXAA-induced serum TNF activity by thalidomide and its analogues. Individual mice were treated with DMXAA (50 mg $kg^{-1}$) alone or together with thalidomide, PP, PEP or PEFP at the indicated doses, or with drug alone. Sera were collected 2 h later and assayed for TNF activity. The control activity for PP and PEP was 3393 units and that for PEFP and thalidomide was 2440 units understood. In cultured cells, thalidomide can either inhibit or enhance TNF production, depending on the conditions and the cell type. TNF production by HL-60 cells in response to phorbol esters was increased by co-incubation with thalidomide, as well as by the phthalimide analogues used in this study (Nishimura et al, 1994). Thalidomide also enhanced TNF synthesis (in response to lipopolysaccharide) in THP-1 cells and in human mononuclear cells enriched for adherent cells, but inhibited TNF synthesis in cultures of unfractionated peripheral blood cells (Shannon and Sandoval, 1996). We do not yet know whether these mechanisms will apply in vivo or whether other mechanisms are involved. We are currently investigating whether these drugs affect the in vivo pharmacokinetics of DMXAA.

The observation that lowering of serum TNF levels is associated with improving the anti-tumour activity of DMXAA appears to contradict previous studies that show that antibodies to TNF inhibit tumour vascular collapse and ameliorate FAA-induced anti-tumour action (Mahadevan et al, 1990; Pratesi et al, 1990). In another tumour model, we have shown that antibodies to TNF partly inhibit the action of DMXAA, consistent with the above observations (WL Browne et al, manuscript in preparation). In recent studies, we have shown that TNF is produced in response to DMXAA in tumour tissue and that co-administration of thalidomide, while reducing serum TNF, does not reduce tumour-associated TNF (WL Browne et al, manuscript in preparation). Further studies are now in progress to determine the mechanism by which

**Figure 7** Effect of thalidomide and its analogues on DMXAA-induced tumour growth inhibition. Colon 38 tumours were measured either untreated (○) or after treatment with DMXAA (30 mg $kg^{-1}$) (●), DMXAA (30 mg $kg^{-1}$) plus thalidomide (10 mg $kg^{-1}$) (◇), DMXAA plus PEP (10 mg $kg^{-1}$) (□), DMXAA plus PEFP (10 mg $kg^{-1}$) (△) or DMXAA plus PP (10 mg $kg^{-1}$) (hexagons)

**Figure 8** Effect of PEFP alone or in combination with DMXAA on tumour growth. Colon 38 tumours were measured either untreated (○) or after treatment with PEFP alone (50 mg $kg^{-1}$) (●), DMXAA alone (■), DMXAA (30 mg $kg^{-1}$) plus PEFP (0.1 mg $kg^{-1}$) (△) or DMXAA (30 mg $kg^{-1}$) plus PEFP (10 mg $kg^{-1}$) (hexagons)

TNF is produced in tumours and to identify the source of the TNF in serum. However, we can conclude from the data obtained so far that the inhibition of serum TNF by thalidomide and the other drugs investigated here does not contradict the hypothesis that TNF mediates the effect of DMXAA in tumours.

In conclusion, we have shown here that pharmacological modulators of TNF production can be applied to reduce serum levels of TNF while increasing the anti-tumour effects of DMXAA. The phthalimide derivatives are more active and potent

**Figure 9** Potentiation of DMXAA-induced tumour growth inhibition by pentoxifylline. (**A**) Growth of Colon 38 tumours in mice with no treatment (●), with pentoxifylline alone (■), DMXAA (30 mg $kg^{-1}$) (▲) or DMXAA together with pentoxifylline (50 mg $kg^{-1}$) (◆). (**B**) A similar experiment using pentoxifylline at a dose of 100 mg $kg^{-1}$

than thalidomide and lack the sedatory effects, presumably through the elimination of the glutarimide substituent of the molecule and interaction with the glutamate receptors in the brain. The co-administration of pharmacological TNF modulators such as these may lead to improved strategies to exploit the novel biological properties of DMXAA in a clinical situation.

### ACKNOWLEDGEMENTS

The authors gratefully acknowledge the excellent technical assistance of Ms Belinda H Gummer in the measurement of anti-tumour responses and of Mr Zhi-Feng Xu in the pentoxifylline studies. This work was supported by the Auckland Division Cancer Society of New Zealand and by the Health Research Council of New Zealand.

### REFERENCES

Baguley BC, Calveley SB, Crowe KK, Fray LM, O'Rourke SA and Smith GP (1989) Comparison of the effects of flavone acetic acid, fostriecin, homoharringtonine and tumour necrosis factor alpha on Colon 38 tumors in mice. *Eur J Cancer Clin Oncol* **25**: 263–269

© Cancer Research Campaign 1998

Casini G and Ferappi M (1964) Preparation of one optical antipode of 2-phthalimidoglutarimide. *Farmaco* **19**: 563–565

Ching L-M and Baguley BC (1987) Induction of natural killer cell activity by the antitumour compound flavone acetic acid (NSC 347512). *Eur J Cancer Clin Oncol* **23**: 1047–1050

Ching L-M, Joseph WR, Zhuang L, Atwell GJ, Rewcastle GR, Denny WA and Baguley BC (1991) Induction of natural killer activity by xanthenone analogues of flavone acetic acid: relation with antitumour activity. *Eur J Cancer* **27**: 79–83

Ching L-M, Xu Z-F, Gummer BH, Palmer BD, Joseph WR and Baguley BC (1995) Effect of thalidomide on tumour necrosis factor production and anti-tumour activity induced by 5,6-dimethylxanthenone-4-acetic acid. *Br J Cancer* **72**: 339–343

D'Amato RJ, Loughnan MS, Flynn E and Folkman J (1994) Thalidomide is an inhibitor of angiogenesis. *Proc Natl Acad Sci USA* **91**: 4082–4085

Fabro S, Smith RL and Williams RT (1967) Toxicity and teratogenicity of optical isomers of thalidomide. *Nature* **215**: 296

Futami H, Eader LA, Back TT, Gruys E, Young HA, Wiltrout RH and Baguley BC (1992) Cytokine induction and therapeutic synergy with interleukin-2 against murine renal and colon cancers by xanthenone-4-acetic acid derivatives. *J Immunother* **12**: 247–255

Han J, Thompson P and Beutler B (1990) Dexamethasone and pentoxifylline inhibit endotoxin-induced cachectin/tumor necrosis factor synthesis at separate points in the signaling pathway. *J Exp Med* **172**: 391–394

Mace KF, Hornung RL, Wiltrout RH and Young HA (1990) Correlation between *in vivo* induction of cytokine gene expression by flavone acetic acid and strict dose dependency and therapeutic efficacy against murine renal cancer. *Cancer Res* **50**: 1742–1747

Mahadevan V, Malik STA, Meager A, Fiers W, Lewis GP and Hart IR (1990) Role of tumor necrosis factor in flavone acetic acid-induced tumor vasculature shutdown. *Cancer Res* **50**: 5537–5542

Moreira AL, Sampaio EP, Zmuidzinas A, Frindt P, Smith KA and Kaplan G (1993) Thalidomide exerts its inhibitory action on tumour necrosis factor alpha by enhancing mRNA degradation. *J Exp Med* **177**: 1675–1680

Nishimura K, Hashimoto Y and Iwasaki S (1994) Enhancement of phorbol ester-induced production of tumor necrosis factor alpha by thalidomide. *Biochem Biophys Res Commun* **199**: 455–460

Noel P, Nelson S, Bokulic R, Bagby G, Lippton H, Lipscomb G and Summer W (1990) Pentoxifylline inhibits lipopolysaccharide-induced serum tumor necrosis factor and mortality. *Life Sci* **47**: 1023–1029

Philpott M, Baguley BC and Ching L-M (1995) Induction of tumour necrosis factor-alpha by single and repeated doses of the antitumour agent 5,6-dimethylxanthenone-4-acetic acid. *Cancer Chemother Pharmacol* **36**: 143–148

Pratesi G, Rodolfo M, Rovetta G and Parmiani G (1990) Role of T cells and tumour necrosis factor in antitumour activity and toxicity of flavone acetic acid. *Eur J Cancer* **26**: 1079–1083

Rewcastle GW, Atwell GJ, Baguley BC, Calveley SB and Denny WA (1989) Potential antitumor agents. 58. Synthesis and structure-activity relationships of substituted xanthenone-4-acetic acids active against the Colon 38 tumor *in vivo*. J Med Chem **32**: 793–799

Rewcastle GW, Atwell GJ, Zhuang L, Baguley BC and Denny WA (1991) Potential antitumor agents. 61. Structure-activity relationships for *in vivo* colon-38 activity among disubstituted 9-oxo-9H-xanthene-4-acetic acids. *J Med Chem* **34**: 217–222

Sampaio EP, Sarno EN, Galilly R, Cohn ZA and Kaplan G (1991) Thalidomide selectively inhibits tumor necrosis factor-alpha production by stimulated human monocytes. *J Exp Med* **173**: 699–703

Sasaki K, Shibata Y, Hashimoto Y and Iwasaki S (1995) Benzylphthalimides and phenethylphthalimides with thalidomide-like activity on the production of tumor necrosis factor alpha. *Biol Pharmaceut Bull* **18**: 1228–1233

Shannon EJ and Sandoval F (1996) Thalidomide can be either agonistic or antagonistic to LPS evoked synthesis of TNF-alpha by mononuclear cells. *Immunopharmacol Immunotoxicol* **18**: 59–72

Teicher BA, Holden SA, Ara G and Northey D (1993) Response of the FSaII fibrosarcoma to antiangiogenic modulators plus cytotoxic agents. *Anticancer Res* **13**: 2101–2106

Zwi LJ, Baguley BC, Gavin JB and Wilson WR (1989) Blood flow failure as a major determinant in the antitumor action of flavone acetic acid (NSC 347512). *J Natl Cancer Inst* **81**: 1005–1013

Zwi LJ, Baguley BC, Gavin JB and Wilson WR (1994) Correlation between immune and vascular activities of xanthenone acetic acid antitumor agents. *Oncol Res* **6**: 79–85

© Cancer Research Campaign 1998